# Modelling Spatial Scales of Dose Deposition and Radiolysis Products from Gold Nanoparticle Sensitisation of Proton Therapy in A Cell: From Intracellular Structures to Adjacent Cells

**DOI:** 10.3390/ijms21124431

**Published:** 2020-06-22

**Authors:** Dylan Peukert, Ivan Kempson, Michael Douglass, Eva Bezak

**Affiliations:** 1 Future Industries Institute, University of South Australia, Mawson Lakes, SA 5095, Australia; ivan.kempson@unisa.edu.au; 2 Division of ITEE, University of South Australia, Mawson Lakes, SA 5095, Australia; 3 Department of Medical Physics, Royal Adelaide Hospital, Adelaide, SA 5000, Australia; michael.douglass@sa.gov.au; 4 Department of Physics, University of Adelaide, Adelaide, SA 5005, Australia; eva.bezak@unisa.edu.au; 5 Cancer Research Institute and School of Health Sciences, University of South Australia, Adelaide, SA 5005, Australia

**Keywords:** Monte Carlo, radiosensitisation, nanoparticles, proton therapy, radiolysis, targeted enhancement, cell model

## Abstract

Gold nanoparticle (GNP) enhanced proton therapy is a promising treatment concept offering increased therapeutic effect. It has been demonstrated in experiments which provided indications that reactive species play a major role. Simulations of the radiolysis yield from GNPs within a cell model were performed using the Geant4 toolkit. The effect of GNP cluster size, distribution and number, cell and nuclear membrane absorption and intercellular yields were evaluated. It was found that clusters distributed near the nucleus increased the nucleus yield by 91% while reducing the cytoplasm yield by 7% relative to a disperse distribution. Smaller cluster sizes increased the yield, 200 nm clusters had nucleus and cytoplasm yields 117% and 35% greater than 500 nm clusters. Nuclear membrane absorption reduced the cytoplasm and nucleus yields by 8% and 35% respectively to a permeable membrane. Intercellular enhancement was negligible. Smaller GNP clusters delivered near sub-cellular targets maximise radiosensitisation. Nuclear membrane absorption reduces the nucleus yield, but can damage the membrane providing another potential pathway for biological effect. The minimal effect on adjacent cells demonstrates that GNPs provide a targeted enhancement for proton therapy, only effecting cells with GNPs internalised. The provided quantitative data will aid further experiments and clinical trials.

## 1. Introduction

Radiotherapy is a modality commonly used in the treatment of cancer. Proton therapy has advantages over conventional megavoltage photon therapy. These advantages are due to the nature of the particles’ interactions with the material it is passing through. Unlike photons, protons are a directly ionising radiation that primarily interact via ionisation interactions with the medium. In ionisation reactions, the energy deposited along the path of the radiation increases as the particle slows down. Accordingly, as an incident proton passes through a medium and loses energy, the proton slows down and the rate of energy loss increases producing a peak in energy deposition prior to the proton stopping known as the Bragg peak. The primary advantage of proton therapy over conventional photon therapy is the superior sparing of healthy tissue. In proton therapy it is possible, by selecting the incident proton energies, to distribute Bragg peaks throughout the target volume. This enables the delivery of a highly conformal dose to the target volume while significantly sparing healthy tissue around the target volume.

The use of radiosensitisers, agents which increase the biological effect of radiation, is a potential technique for improving radiotherapy outcomes. The preferential delivery of radiosensitisers to the tumour can increase the separation of the tumour control and normal tissue complication probabilities for a given dose, allowing a greater therapeutic ratio to be achieved. Gold nanoparticles (GNPs) have desirable characteristics for use as radiosensitisers; they have good biological compatibility and a high Z (atomic number). Additionally, GNPs passively accumulate in tumour tissue preferentially compared to healthy tissue via the enhanced permeability and retention (EPR) effect [1,2]. GNPs can also have extensive surface functionalisation allowing the potential for targeted delivery. GNPs were thought to be effective radiosensitisers only for radiation types with interactions with a dependence on Z such as photoelectric and pair production interactions for photons. As the photoelectric interactions that occur at lower photon energies have a much stronger Z dependence than pair production interactions that occur at higher photon energies, GNPs have a greater radiosensitising effect for kV photons than MV photons. As protons primarily interact via ionisation which has a weak dependence on Z, GNPs were originally not expected to be effective for enhancing proton therapy. However, experimental observations have contradicted this expectation with GNP enhancement of proton therapy being observed in both in-vitro [3,4,5] and in-vivo [6,7] experiments. Experiments have also provided indications that a large proportion of the observed enhancement is due to increased indirect damage by reactive species produced via radiolysis [5,8]. A review of chemical mechanisms for nanoparticle radiosensitisation has been given by Howard et al. [9].

The use of GNP radiosensitisers with proton therapy offers the prospect of an advanced treatment combining the excellent sparing of healthy tissue of proton therapy with an increased biological effect within the tumour from the GNPs. However, for this novel treatment to progress to clinical use it is essential to gain a greater understanding of the enhancement mechanisms and the biological targets effected within a cell in order to optimise nanoparticle design to ensure an effective treatment. Due to the time and expense required it would be difficult for one group to perform sufficient experimental observations to optimise nanoparticle design. Combined with the difficulty in comparing experiments between groups due to the many possible variables such as cell type, nanoparticle production and functionalisation methods and radiation source, this suggests that the development of a model of the biological effect due to GNPs for proton therapy is desirable to aid our understanding of the underlying mechanism of the observed enhancement and optimise nanoparticle design.

Several theoretical studies have endeavoured to explain the enhanced biological effect due to GNPs for proton therapy. Techniques successfully used to model GNP enhancement of conventional photon therapy were applied to GNP enhanced proton therapy. Simulations of macroscopic dose enhancement performed by Cho [10] were able to model GNP enhancement for kV photons; however, macroscopic simulations by Cho et al. [11] and calculations of the macroscopic dose from the dose distribution around a single GNP by Kwon et al. [12] found that even small increases in the macroscopic dose required extremely high GNP concentrations greater than the concentrations used in experiments and what is clinically feasible. Simulations of the dose enhancement to the nucleus for a 2D cell model utilising the local effect model (LEM) to calculate the additional biological effect from the presence of the GNPs performed by McMahon et al. [13] were able to model GNP enhancement for both kV and MV photons. However, when similar simulation techniques were used to model proton therapy by Lin et al. [14], it was found that for GNPs distributed within the cytoplasm no enhancement was observed. To achieve a non-negligible enhancement with the model it was required that GNPs were present within the nucleus. Simulations of direct damage to the nuclear DNA by Sotiropoulos et al. [15] also found no enhancement from GNPs within the cytoplasm for proton therapy. Experimental observations of the GNP distribution in cells [5,16,17,18] show that while GNPs can enter the cytoplasm they do not typically enter the nucleus. The inability to model enhancement for protons when GNPs are within the cytoplasm is likely due to faster radial dose falloff around a GNP irradiated with protons than for incident photons [19]. Furthermore, experiments indicate indirect damage from reactive species can play a significant role. These observations imply that incorporating radiolysis into simulations is essential to model GNP enhanced proton therapy. An overview of experimental observations and simulations of GNP enhanced radiotherapy has been given by several reviews [20,21,22].

A few previous studies have performed stochastic modelling of the radiolysis yield enhancement from GNPs for proton therapy. Tran et al. [23] used the Geant4 Monte Carlo toolkit [24,25,26] to model the radial dose and radiolysis yield enhancement around a single 50 nm GNP irradiated with a proton beam. It was found that while the dose and initial (up to 10 ns) radiolysis yield enhancement were similar, the radial enhancement ratios increased with greater distance from the GNP until reaching a plateau at an enhancement value of 14 for 10 MeV and higher proton energies. The radial enhancement then reduced at distances beyond the maximum range of secondary electrons produced from the GNP. At later times in the chemistry stage, the enhancement closer to the GNP was reduced due to the reactions and diffusion of the reactive species. Hespeels et al. [27] used similar methods to model the radial dose and yield enhancement around 5 and 10 nm GNPs for 2 MeV protons using the Geant4 Monte Carlo toolkit [24,25,26] with both default ionisation models and their implementation of binary encounter approach models. It was found that the radial yields with the default models were in agreement of those found by Tran et al. and that the enhancement was greater for 5 nm GNPs than for 10 nm GNPs.

In previous work by the current authors, an optimisation study of GNP characteristics was performed to maximise the radiolysis yield enhancement [28]. This study was the first application of the simulation framework developed by the authors using the Geant4 Monte Carlo toolkit [24,25,26]. In the simulation framework, the simulation was performed in two stages. In the first, the interactions of the incident protons within the GNPs was modelled and secondary electrons escaping the GNP were modelled in a phase space file. This was then used as an input for the second stage which modelled the dose and radiolysis species yield in the surrounding water. The dose and yield enhancement for the GNP was determined relative to an equivalent WNP. It was found that intermediate nanoparticle sizes of 10–25 nm provided the optimal balance between gold mass and limiting self-absorption. Thin nanoparticle coatings further minimised the loss of the enhancement from the gold core. The 3D distribution of the reactive species yield and the effects of nanoparticle proximity and clustering was also modelled [29]. This investigation was performed using a later version of the simulation framework that had additional features incorporated. The simulation was able to score the secondary electrons leaving multiple nanoparticles in a single-phase space file. The combined file accounted for the potential for the electrons to be absorbed by other GNPs as well as the GNP the electron was emitted from. The ability to score the full 3D distribution of the dose and reactive species yield over time was also added to the simulation. It was determined that the reactive species’ distribution around a GNP had a greater range and was less forward distributed than the equivalent water nanoparticle (WNP). This caused a larger relative enhancement at greater distances from the nanoparticle and in the backward direction where a larger proportion of the reactive species were produced for the GNP compared to the WNP. It was found that having nanoparticles in proximity to each other resulted in a loss of energy deposited and total reactive species yield from increased absorption of secondary electrons. A loss of up to 18% was observed for two 15 nm GNPs nanoparticles while the absorption loss for a 500 nm cluster of 15 nm GNPs was 60%. While clusters resulted in a large loss of 60% due to absorption, they also caused highly concentrated reactive species yields in the vicinity of the cluster. Rudek [30] et al. modelled the radiolysis yield per deposited energy (G (species/100 eV)) within a cell model for various GNP distributions with GNPs treated as reactive species scavengers. Note that as the radiolysis of both the primary protons and the secondary electrons emitted from GNPs was modelled due to computational constraints a very small cell model size of 1.5 µm was used in their work which is not representative of a typical cell. Additionally, G is a measure of the efficiency of reactive species production per energy deposited which is primarily dependant on the linear energy transfer (LET) of the incident radiation and provides limited additional information.

In this work, the simulation framework previously developed by the authors [28,29] was used to model the enhancement from GNP distributions with a cell model. The cell model simulations were used to investigate (a) the effect of the size, distribution and number of GNP clusters on the absolute radiolysis yield within cellular components, (b) the effect of the reactive species permeability of the nuclear and cellular membranes on the reactive species yield as well as the distribution of reactive species stopped within the membrane and (c) the reactive species yield in a second cell adjacent to a cell containing GNPs from secondary electrons emitted from the GNPs for various cell separations. The effect of GNP cluster characteristics on the absolute radiolysis yield within the components of a representative cell model was considered for the first time. The effect of membrane permeability to reactive species on the yield within cellular components as well as the distribution of the reactive species within the membrane were considered for the first time. Inter-cellular GNP enhancement effects were also evaluated for the first time. The results of these simulations provide the first quantitative data for many of the investigated effects which will aid identifying potential biological targets for GNP enhancement of proton therapy and optimisation of the cellular GNP distribution to maximise the enhancement effect for the biological targets.

## 2. Results

### 2.1. GNP Cellular Distribution

**Figure 1:**
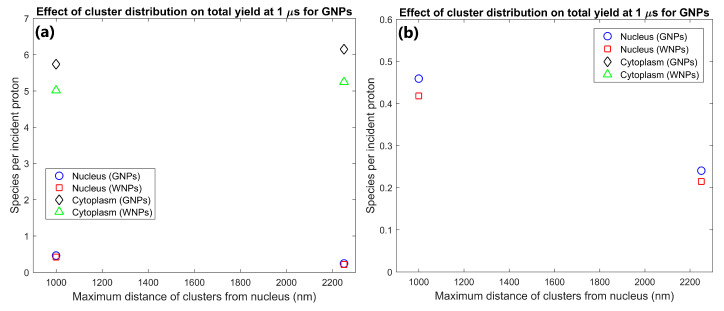
(**a**) Effect of proximity of GNP and WNP cluster distributions to the nucleus on reactive species yield at 1 µs from secondary electrons for cells with 1000 clusters of 200 nm diameter within the cytoplasm. (**b**) Highlight of nucleus yields shown in (a).

The effect of the proximity of the GNP cluster distribution to the nucleus on the radiolysis yield at 1 µs within the nucleus and cytoplasm of the cell is shown in Figure 1. The GNP distributions with clusters distributed within 1 µm of the nuclear membrane (consistent with experimental observations [16]) resulted in a radiolysis yield within the nucleus 91% higher than for GNP clusters distributed throughout the cytoplasm. However, having the GNPs clustered near the nucleus also reduced the cytoplasm yield by 7% compared to GNP clusters being distributed throughout the cytoplasm.

**Figure 2:**
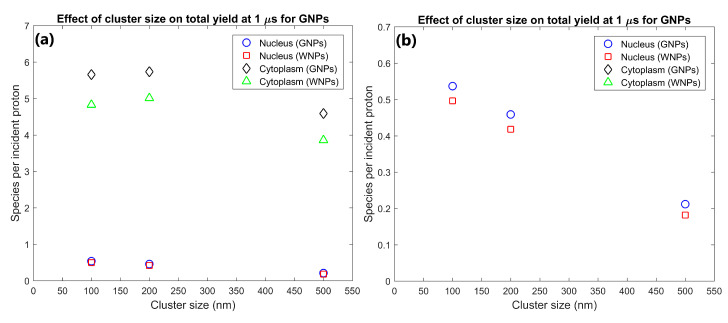
(**a**) Effect of GNP and WNP cluster size on reactive species yield at 1 µs from secondary electrons for cells with 70 clusters of 500 nm diameter, 1000 clusters of 200 nm diameter and 3400 clusters of 100 nm diameter within the cytoplasm. The number of clusters was chosen so that each cell contained 10^5^ 15 nm nanoparticles. (**b**) Highlight of nucleus yields shown in (a).

The effect of GNP cluster size on the radiolysis yield at 1 µs within the nucleus and cytoplasm of the cell is shown in Figure 2. The cytoplasm yield is maximised for 100 and 200 nm clusters and is reduced for larger clusters while the nucleus yield is higher for smaller cluster sizes. The yield within the cytoplasm and nucleus for 200 nm clusters were 25 and 117% greater respectively than the yields for 500 nm clusters. The yield within the cytoplasm and nucleus for 100 nm clusters were 23% and 153% greater respectively than the yields for 500 nm clusters.

**Figure 3:**
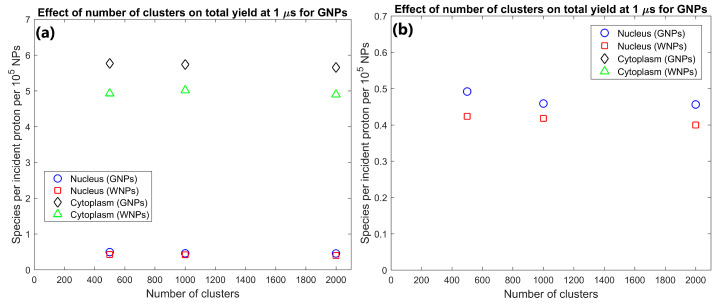
(**a**) Effect of number of gold nanoparticle (GNP) and water nanoparticle (WNP) clusters on reactive species yield at 1 µs from secondary electrons for cells with 500–2000 clusters of 200 nm diameter within the cytoplasm. Yield per 1000 clusters is shown. (**b**) Highlight of nucleus yields shown in (a).

The effect of the number of GNP clusters on the radiolysis yield at 1 µs per GNP in the cytoplasm and nucleus is shown in Figure 3. Overall, the yield per GNP is mostly constant indicating an effective scaling of yield with GNP concentration within the cell. There is a slight reduction in the yield per GNP from 500 to 1000 clusters of 6.7% for the nucleus and 1.4% from 1000 to 2000 clusters for the cytoplasm.

The dose and total radiolysis yield at 1 µs within the nucleus and cytoplasm as well as the dose enhancement factor (DEF) and radiolysis enhancement factor (REF) for GNP cellular distribution simulations shown in Table 1. A summary of the key results is also shown in the table. For all cluster distributions simulated an enhancement of between 7–19% is observed in the energy deposited and reactive species yield for GNPs compared to WNPs.

Cross section slices of the full 3D reactive species distribution within the cell model for 1000 clusters with a diameter of 200 nm clustered near the nuclear membrane are shown in Supplementary Video 1.

### 2.2. Absorption in Nuclear Membrane

**Figure 4:**
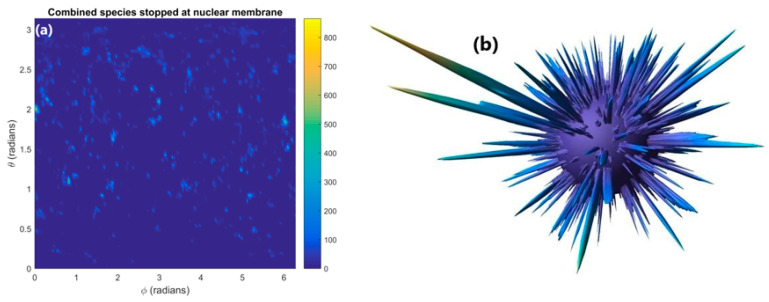
(**a**) The distribution of reactive species stopped within the nuclear membrane. (**b**) A 3D plot with the concentration of reactive species indicated by the distance from the surface of the sphere of the nuclear membrane.

The distribution of reactive species stopped within the nuclear membrane is shown in Figure 4a. The distribution is highly heterogenous with numerous concentrated hotspots where many reactive species have been absorbed in the membrane. These concentrated hotspots are clearly shown as intensity spikes emanating from the surface of the nuclear membrane in the 3D diagram shown in Figure 4b. Much of the surface area of the nuclear membrane has no or very few reactive species being absorbed. By considering the nuclear membrane to be either 0 or 100% permeable the effect of absorption in the nuclear membrane on the reactive species yield at 1 µs in the nucleus and cytoplasm was determined. Absorption of reactive species within the nuclear membrane reduces the reactive species yield at 1 µs relative to full diffusion through the membrane by 8 and 35% for the cytoplasm and nucleus respectively.

### 2.3. Inter-Cellular Effects and Cell Membrane Absorption

**Figure 5:**
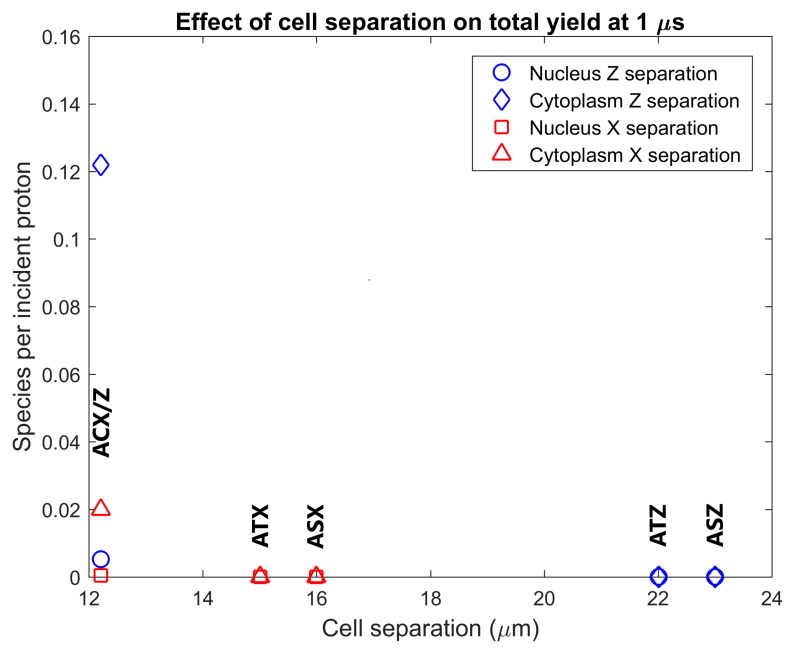
Effect of separation between the centre of the nuclei of the cells on the radiolysis yield at 1 µs in the nucleus and cytoplasm of the adjacent cell from electrons emitted by GNPs within the primary cell. Results are shown for separations parallel to the proton beam (along the Z axis) and perpendicular to the proton beam (along the X axis). The separations along each axis were chosen representing from lowest to highest respectively cells packed together causing a flattening of the cells just beyond the GNP distribution radius, adjacent cells with the membranes in close contact but not compressed together and adjacent cells with a 1 µm gap between the cell membranes.

The radiolysis yield at 1 µs within the cytoplasm and nucleus of adjacent cells from secondary electrons emitted from GNPs in the primary cell was determined. The dependence of the adjacent cell yield on the separation between the centres of the nuclei of the primary and adjacent cell for separations perpendicular and parallel to the proton beam is shown in Figure 5. Reactive species are observed in the cytoplasm and nucleus of the adjacent cell for tightly packed cells, while no, or minimal, reactive species yield is observed in adjacent cells for cells just touching and for greater separations. The yield for an adjacent cell positioned forward of the primary cell along the beam path is over 6 times greater than the yield for an adjacent cell separated perpendicular to the proton beam for tightly packed cells.

**Figure 6:**
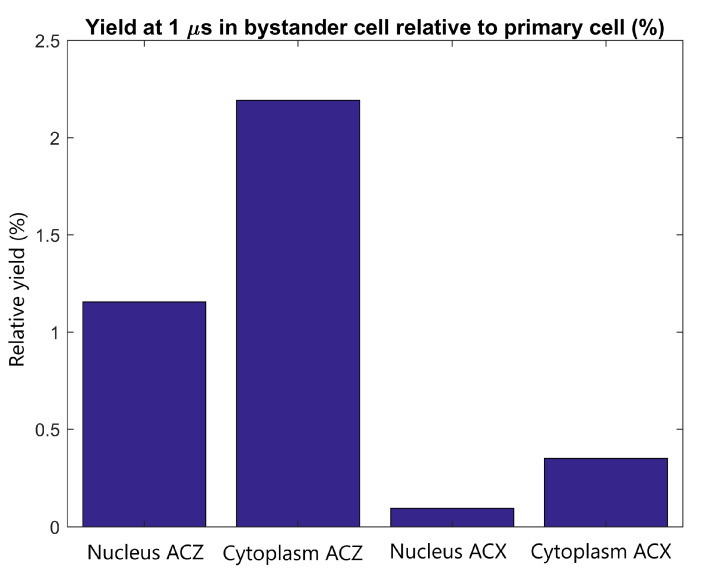
The radiolysis yield at 1 µs in the nucleus and cytoplasm of the adjacent cell relative to the yield for the primary cell from electrons emitted by GNPs within the primary cell. Results are shown for tightly packed cells with separations parallel to the proton beam (along the *Z* axis) and perpendicular to the proton beam (along the *X* axis).

The radiolysis yield at 1 µs in the cytoplasm and nucleus of the adjacent cell relative to the yield in the cytoplasm and nucleus of the primary cell from secondary electrons emitted by GNPs within the primary cell is shown in Figure 6. For a tightly packed adjacent cell forward of the primary cell along the beam path, the yield in the cytoplasm and nucleus is 2.2% and 1.2% of the cytoplasm and nucleus yield of the primary cell respectively. For an adjacent cell separated from the primary cell perpendicular to the proton beam the relative yield is less than 0.5%.

**Figure 7:**
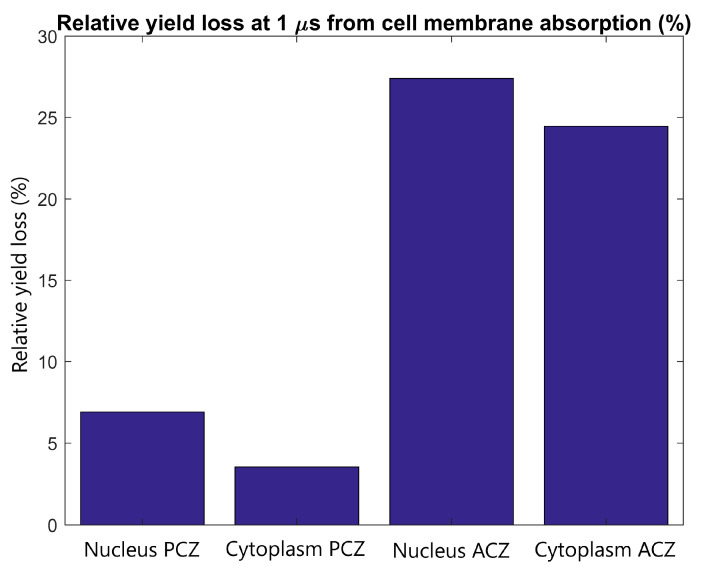
The relative radiolysis yield loss at 1 µs in the nucleus and cytoplasm of the adjacent and primary cell from electrons emitted by GNPs within the primary cell with full absorption at the cell membrane compared to the yield for without absorption. Results are shown for tightly packed cells separated parallel to the proton beam (along the *Z* axis).

The effect of cell membrane absorption on the radiolysis yield at 1 µs in the primary and adjacent cells was determined for tightly packed cells with the adjacent cell separated in the direction of the proton beam. The reduction in the reactive species yield at 1 µs in the nucleus and cytoplasm of the primary and adjacent cells is shown in Figure 7. It was found that the absorption of reactive species within the cellular membrane reduces the yield at the end of the chemistry stage by 3-8% and 25-30% for the primary and adjacent cell respectively.

## 3. Discussion

### 3.1. GNP Cellular Distribution

The short range of secondary electrons and their susceptibility to absorption seen in earlier cluster simulations [29] leads to the distribution of GNPs within the cell having a major impact on the yield within intra-cellular components.

The effect of the proximity of the GNP clusters on the nucleus and cytoplasm yields shown in Figure 1 shows that GNP clusters in close proximity to the nuclear membrane are essential to maximise the radiosensitisation effect within the nucleus. The nucleus yield is increased by 91% by having GNP clusters within 1 µm of the nuclear membrane instead of being distributed within a larger region of the cytoplasm. This is due to the short range of low energy secondary electrons emitted from the GNPs, meaning that to achieve a large dose and yield to the cell nucleus, the GNPs must be as close to the nucleus as possible. Note that this increase in the nucleus yield is at the expense of a smaller relative but larger absolute reduction in the cytoplasm yield. This is due to a combination of a greater proportion of the dose and resulting radiolysis yield falling within the nucleus instead of the cytoplasm and an increase in inter-cluster absorption from the reduced average separation of the GNP clusters distributed in a smaller volume. The large increase in the reactive species yield within the nucleus from having GNPs in the perinuclear region of the cytoplasm quantified in this study demonstrates that for effective enhancement of a biological target the GNPs used should be designed to accumulate within a cell as close to the target as possible.

The dependence of the nucleus and cytoplasm yield on the cluster size shown in Figure 2 indicates that increased intra-cluster absorption for larger cluster sizes can significantly reduce the cell component yields. Larger clusters containing more GNPs result in secondary electrons produced within a GNP within the cluster being more likely to interact with other GNPs within the cluster resulting in absorption losses. This reduces both the total energy deposited in water and the energy and hence range of secondary electrons leaving the cluster. The large absorption losses for larger cluster sizes indicate that to maximise the dose and radiolysis enhancement around the GNP clusters, the GNPs should be designed to avoid accumulating into clusters larger than a few hundred nanometres in diameter. While both the nucleus and cytoplasm yields are reduced for larger cluster sizes, the relative reduction compared to 100 nm clusters is greater and begins at smaller cluster sizes for the nucleus than the cytoplasm. This is due to the range reduction of the secondary electrons resulting in fewer secondary electrons being able to reach the nucleus. This results in a greater proportion of the energy of secondary electrons being deposited within the cytoplasm, increasing the effect of absorption loss on the nucleus yield while reducing the effect on the cytoplasm yield.

The effect of the number of GNP clusters on the yield per GNP shown in Figure 3 demonstrates that as the value is reasonably constant there is no saturation of the yield increase from greater numbers of GNPs within a cell for clinically feasible concentrations. Only a slight decrease is observed for higher concentrations from the increased inter-cluster absorption from the reduced average separation of clusters. Similarly, to the intra-cluster absorption, the reduced range of the secondary electrons causes the reduction to occur earlier and to a larger degree for the nucleus yield compared to the cytoplasm yield. The low levels of inter-cluster absorption even for higher GNP concentrations indicates that the radial dose and reactive species yield distribution around each cluster can be reasonably approximated by the radial dose yield from single cluster simulations such as those investigated by the authors in a previous study [29]. The low levels of inter-cluster absorption also demonstrates that increasing the GNP content within a cell will increase the enhancement effect. For clinically relevant GNP concentrations, there is no potential for the high GNP concentrations to reduce the enhancement from the GNPs or induce a radioprotective effect from absorption of radiation within the GNPs.

The DEFs and REFs shown in Table 1 show that for all cluster distributions considered the energy deposited and radiolysis yield within the nucleus and cytoplasm from secondary electrons emitted from the nanoparticles was increased by between 7% and 19% for GNPs compared to the equivalent WNPs. However, when the contribution from the incident primary protons is taken into account the macroscale enhancement is negligible. This suggests that the primary benefit of GNPs is not a macroscale increase in dose and reactive species yield but instead the production of regions of highly concentrated reactive species yield and energy deposition in proximity to the GNP cluster as shown in Supplementary Video 1. These highly concentrated regions of energy deposition and reactive species yield have the potential for enhanced biological effect.

### 3.2. Absorption in Nuclear Membrane

The reduction in reactive species yield at 1 µs from absorption in the nuclear membrane shown in Figure 4 indicates that absorption in the nuclear membrane can significantly reduce the radiolysis yield, especially within the nucleus. The loss in yield for the nucleus is considerably greater than the loss in the cytoplasm due to more of the nucleus’ volume being within the range of reactive species diffusion of the nuclear membrane. This means that reactive species within the nucleus are more likely to be able to reach the nuclear membrane and be absorbed than reactive species in the cytoplasm. Additionally, due to the greater reactive species’ concentration near the GNPs within the cytoplasm more reactive species diffuse from the cytoplasm to the nucleus than diffuse from the nucleus to the cytoplasm. This causes the loss of species being able to diffuse between the cytoplasm and the nucleus to have a greater impact on the yield within the nucleus.

The 35% reduction in the reactive species yield in the nucleus at 1 µs indicates that absorption within the nuclear membrane can cause a large reduction in indirect damage to DNA within the nucleus from the presence of GNPs. However, the dense concentrations of reactive species stopped within the nuclear membrane have the potential to rupture the nuclear membrane allowing oxidising species present within the cytoplasm to enter the nucleus. This damage pathway may offset the reduction in reactive species yield within the nucleus.

### 3.3. Inter-Cellular Effects and Cell Membrane Absorption

The radiolysis yield in adjacent cells from solely the secondary electrons emitted by GNPs within the primary cell shown in Figure 5 shows that the yields for cells separated from, or just touching, the primary cell are negligible. A non-negligible yield is only observed for adjacent cells compressed against the primary cell representing a tightly packed tissue. This is due to the limited range of the low energy secondary electrons emitted from the GNPs; the tightly packed cells have the GNP distribution considerably closer to the interface between the cells allowing secondary electrons to reach the adjacent cell. The yield in adjacent cells forward of the primary cell along the beam path is larger than the yield in an adjacent yield separated perpendicular to the beam path, due to the forward bias in the production of secondary electrons within the GNPs.

The yields in the adjacent cell from secondary electrons emitted from GNPs in the primary cell relative to the primary cell yields shown in Figure 6 demonstrate that the yield in the adjacent cell, even in an ideal tightly packed geometry, is small compared to the yield in the primary cell. Even for the ideal scenario of an adjacent cell forward of the primary cell the yield is less than 2.5% of that in the primary cell while for adjacent cells perpendicular to the beam the yield is less than 0.5% of that of the primary yield. The close proximity of tightly packed cells allows for some of the low energy electrons emitted from the GNPs in the primary cell to reach the adjacent cell. However, due to the short range of the electrons primarily only GNP clusters nearest the adjacent cell contribute to the dose and yield in the adjacent cell.

Enhancement for adjacent cells from GNPs within a cell is limited, a very small enhancement of less than 2.5% that of the primary cell is seen and only for tightly packed cells. The enhancement from GNPs is predominantly localised within the cells containing the GNPs. Therefore, GNP enhancement of proton therapy is a targeted enhancement which only affects cells which uptake GNPs. This is beneficial, as healthy cells adjacent to cancer cells receive minimal enhancement from GNP treatments provided that the GNP design achieves preferential uptake by cancer cells. Note that while the enhancement is targeted, nearby healthy cells will still receive the full unenhanced radiation dose from the proton therapy treatment. The targeted enhancement combined with the low inter-cluster absorption indicates that, provided the GNPs preferentially accumulate in cancerous cells compared to surrounding healthy tissue, it is desirable to maximise the GNP concentration within the cancer cells. This will increase the enhancement effect only within the cancerous cells and not surrounding healthy tissue.

The yield loss in the primary and adjacent cells from absorption in the cell membrane shown in Figure 7 indicates that the primary cell has only a small loss of 4–8% which is partially offset by the potential for clusters of reactive species to damage the cell membrane. While a larger relative loss of 25–30% is observed in the adjacent cell, the absolute loss is low due to the minimal yield in the adjacent cell.

### 3.4. Validation and Limitations

Validation of the Geant4-DNA chemistry models with experimental observations of water radiolysis yields [31] demonstrated that the yields of individual reactive species for lower LET radiation were within 20% of experimental measurements with most within 10%. This indicates that the chemistry models used accurately model the radiolysis yields over the time of the chemistry stage. The Geant4-DNA chemistry models were also found to account well for the relative change in yield due to changes in the energy deposition distribution with the change in yield with LET being within 5% of experimental measurements. This indicates that the models can accurately simulate the effects of differing energy deposition distributions on the resulting rates of the diffusion limited reactions and hence that the diffusion is accurately modelled. Unfortunately, experimental observations of the reactive species yield distribution within a cell resulting from GNP enhanced radiotherapy are not available for direct validation. However, the validation demonstrating the accuracy of reactive species yields, diffusion and reaction rates for the Geant4-DNA chemistry models [31] indicates that the models can be used to model the yield distribution resulting from various GNP cluster distributions and the diffusion of the reactive species over time.

In this work the effects of reactive species interaction with the GNPs was not considered, GNPs were treated as non-interactive with the reactive species. Only the reactive species produced by secondary electrons emitted from nanoparticles were modelled to allow a representative cell model size within reasonable computational requirements. As the entire Geant4-DNA physics and chemistry models are currently only available for water the components of the cell model were modelled as consisting of water. While water is a good model for soft tissue, there will be a slight impact from using water instead of soft tissue within the simulation. Biological endpoints such as cell survival probability were not modelled in this work.

## 4. Materials and Methods

The Geant4 Monte Carlo toolkit [24,25,26] (Version 10.5) was used for the 3D cell model simulations presented in this work. The simulations were performed in two stages. In the first stage of the simulation, the full cellular geometry with various GNP cluster sizes and distributions was modelled. A proton beam was modelled incident on the cell model, and the physical interactions of the protons and resulting secondary particles were modelled using the Livermore low energy physics models. The dose distribution in the cell components; the cell membrane, cytoplasm, nuclear membrane and nucleus were scored. Additionally, secondary electrons were recorded in a phase space file when the electron either leaved or entered a GNP. In the second stage of the simulation, the interactions of secondary electrons leaving the GNPs within the surrounding water, the resulting water radiolysis and the diffusion and reactions of the produced reactive species were modelled using the Geant4-DNA very low energy physics and chemistry models [31,32,33,34,35,36,37] The distribution of the dose and reactive species yield over time was scored for the duration of the simulation from the commencement of irradiation to 1 µs post irradiation. The dose and reactive species yields presented are the dose and yield per incident proton. This scaling per incident proton enables the determination of the dose and yield for any incident proton flux of interest. The parameters used in the simulations performed in this work are presented in Table 2. The simulations were performed using a 3.4 GHz AMD Ryzen 1950X CPU with 16 physical cores and 32 computational threads with 32 GB of RAM. The computation time for each simulation was 6–24 h for the first stage of the simulation and 16–24 h for the second stage of the simulation.

**Figure 8:**
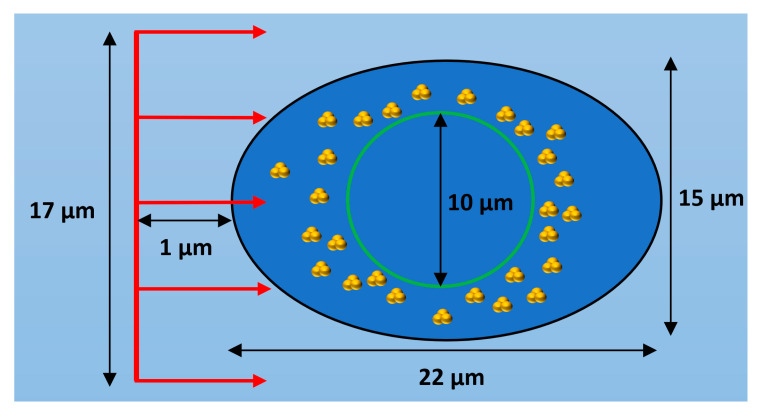
Diagram of cell model showing a cross section of the ellipsoid cell with a black outline with the spherical nucleus shown by a green outline and GNP clusters distributed within the cytoplasm. The incident proton beam is shown in red and is travelling in the positive Z direction. Not to scale.

The geometry of the first simulation stage consisted of a 25 by 25 by 25 µm^3^ water volume which contained a cell model with cell and nucleus dimensions based on observations of HeLa cells [16]. The cell model geometry is shown in Figure 8 and consists of a cell boundary defined by a general ellipsoid with one major axis of 22 µm in length, two minor axes both of 15 µm and a nucleus boundary defined by a sphere with a diameter of 10 µm. Both the cell and nuclear membranes had thicknesses of 20 nm from the cell boundary and nucleus radius respectively. The region between the cell membrane and nuclear membrane defined the cytoplasm volume while the region within the nuclear membrane defined the nucleus volume. All cell components were modelled as consisting of water, this was chosen as it is the only medium for which the full Geant4-DNA physics and chemistry models are available. The nanoparticle geometry consisted of clusters of 15 nm nanoparticles constructed from in vitro measurements of GNP clustering within HeLa cells [16]. Bare 15 nm GNPs were chosen to be modelled as a previous optimisation study by the authors [28] showed that 15 nm is an optimal GNP size to maximise enhancement, providing a balance of gold mass and limited self-absorption of secondary electrons within the GNPs. The study also showed that thicker nanoparticle coatings reduced the enhancement by absorbing secondary electrons produced within the gold core preventing their contribution to the dose and reactive species yield outside of the nanoparticle. Therefore, bare GNPs were modelled to represent the maximum potential radiosensitisation to consider a best-case scenario. The clusters were randomly distributed within either the entire cytoplasm or a section of it. For most simulations, the clusters were distributed within 1 µm of the nuclear membrane to reproduce the accumulation of GNP clusters near the nuclear membrane observed in HeLa cells [16]. The number of GNPs within the cell model in the simulations performed ranged from 5 × 10^4^ to 2 × 10^5^ representing a range of GNP concentrations observed in various cancer cells [38]. While the simulation geometry is based on HeLa cell measurements the observed distribution of GNPs near the nucleus is frequently observed in measurements of the internal GNP distribution within cells [16,17,18]. Therefore, the results found will be applicable to a wide variety of cells and GNP types.

A circular proton beam with a diameter of 17 µm was modelled incident upon the cell model. The proton source was located 12 µm from the centre of the cell and the initial proton direction was along the Z axis shown in Figure 8. A mono-energetic proton energy of 5 MeV was used as lower proton energies are characteristic of the proton beam within the spread-out Bragg peak (SOBP) which is localised within the tumour in proton therapy treatment. The proton beam size and source position were chosen to ensure a 1 µm water gap between the beam source and the edge of the beam and the cell model. A 1 µm water gap was chosen to ensure a reasonable level of electronic equilibrium while minimising the increase to the simulation time from modelling additional interactions with water outside of the cell.

The effect of the distribution of GNPs within the cytoplasm of a cell on the radiolysis yield in the cytoplasm and the nucleus of the cell was investigated. The effect of cluster size on the radiolysis yield was determined by simulating the radiolysis yield in the nucleus and cytoplasm for 100, 200 and 500 nm diameter clusters containing 34, 100 and 1298 GNPs respectively. The GNPs were modelled in a cluster geometry as this is a common intracellular distribution observed in experiments [16,17,18]. The clustering of GNPs was found to result in high local concentrations of dose and reactive species yield compared to single nanoparticles but also a reduction in the total yield from intra-cluster absorption in a previous work by the authors [29]. The chosen cluster sizes represented the range of clustering and were generated from the clustering observed by Chen et al. [16]. The dependence of the cluster distribution on the yield in the cell components was found by simulating GNP clusters distributed within 1 and 2.25 µm of the nuclear membrane. The majority of the simulations had GNP clusters within the perinuclear region of the cytoplasm as it is a common distribution of GNPs within a cell observed in experiments [16,17,18] which maximises the potential for enhancement of damage to the nucleus. The effect of the number of clusters was investigated by simulating 500, 1000 and 2000 GNP clusters of a diameter of 200 nm within the cell and calculating the radiolysis yield in the nucleus and cytoplasm per GNP to determine if the yield scaled with the number of GNPs. The increase in the energy deposited and radiolysis yield within the cytoplasm and nucleus was determined by calculating the dose enhancement factor (DEF) and the radiolysis enhancement factor (REF). The dose enhancement factor is defined by $DEF=D_{GNP}^{R}/D_{WNP}^{R}$ where $D_{GNP}^{R}$ and $D_{WNP}^{R}$ are the dose from GNP clusters and the equivalent WNP clusters respectively within region R. The radiolysis enhancement factor is defined by $REF=Y_{GNP}^{R}/Y_{WNP}^{R}$ where $Y_{GNP}^{R}$ and $Y_{WNP}^{R}$ are the reactive species yield from GNP clusters and the equivalent WNP clusters respectively within region R. Note that the enhancement factors only consider the dose and yield from secondary electrons emitted by the nanoparticles.

The effect of absorption of reactive species by the nuclear membrane was determined by calculating the radiolysis yield at 1 µs within the nucleus and cytoplasm for both full diffusion of reactive species through the nuclear membrane and complete absorption of reactive species within the membrane. For permeable membranes the modelled reactive species were able to freely pass through the membrane within the simulation, while for absorptive membranes the tracking of reactive species within the simulation was ceased if the species entered the membrane. The position of the final diffusion step that ended in the membrane for absorbed reactive species was recorded. The distribution of reactive species absorbed in the nuclear membrane was also examined to determine the potential for damage to the membrane.

**Figure 9:**
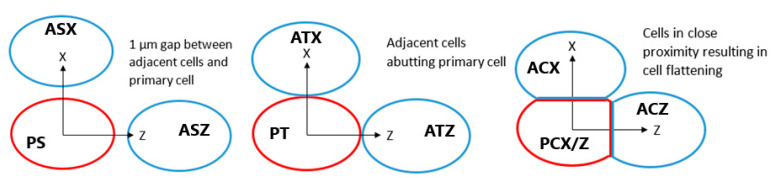
Diagram showing positions of adjacent cells in blue relative to the primary cell containing GNPs in red for separated, touching and compressed cells. Each cell is assigned a label, for the first letter P indicates the primary cell and A indicates the adjacent cell, for the second letter C denotes compressed cells, T denotes touching cells and S indicates separated cells, for the third letter X and Z indicate cells separated along the X and Z axes respectively. Not to scale.

The potential for inter-cellular effects from internalised GNPs was evaluated by modelling the reactive species yield in a cell adjacent to the primary GNP containing cell from secondary electrons emitted by the GNPs in the primary cell. Adjacent cells were modelled tightly packed with the primary cell, just touching the primary cell and separated by 1 µm from the primary cell for separations parallel to, and perpendicular to, the proton beam direction as shown in Figure 9. For the tightly packed cells a planar cut is applied to the outer boundaries of the cell membrane and the cytoplasm at distances of 6.1 µm and 6.08 µm from the centre of the nucleus in the direction of the other cell respectively. These values were chosen so that the compression was such that the GNP distribution reached the flattened cell boundary to maximise the potential for intercellular effects while preserving the 20 nm thickness of the cell membrane. The effect of cell membrane absorption was considered for tightly packed cells separated along the beam as this was the scenario with the greatest inter-cellular effects. The nucleus and cytoplasm yields at 1 µs with membrane absorption relative to the yields with full permeability were determined for the primary and adjacent cells.

The number of primary protons modelled was chosen to be between 2.7 × 10^5^ and 1.24 × 10^6^ to provide a balance between minimising statistical uncertainty and computation time. Repeated simulations showed that for the results presented the relative statistical uncertainty was less than 3% and 0.5% for yields within the nucleus and cytoplasm respectively. The presented dose and yields per incident proton can be scaled to the proton flux for a particular proton therapy treatment of interest or for comparison with experimental observations.

## 5. Conclusions

The simulations performed in this work modelled proton irradiation of a HeLa cell containing GNP clusters to investigate enhancement of both dose deposition and reactive species yields. Modelling reactive species generated from secondary electrons emitted by GNP clusters within the cytoplasm is used to provide mechanistic understanding of how sensitisation to protons occurs. These data also inform how the exploitation of these mechanisms may be maximised. The high absorbability and limited range of the low energy secondary electrons emitted from the GNPs affects the ideal cluster size and distribution. Smaller cluster sizes result in higher yields due to reduced intra-cluster absorption; it was found that larger 500 nm clusters reduced the nucleus yield by 54% compared to 200 nm clusters. Due to the limited range of the enhancement to maximise the effect on a cell component such as the nucleus, the GNPs should be designed to be as near to the component as possible, having GNP clusters within 1 µm of the nucleus increased the nucleus yield by 91% compared to clusters distributed throughout the cytoplasm. It was found that the radiolysis yield scaled efficiently with the number of GNP clusters indicating that for clinically relevant GNP cellular uptake, inter-cluster absorption and shadowing of the proton beam have only a limited effect.

Simulations showed that absorption within the nuclear membrane could significantly reduce the 1 µs yield especially for the nucleus yield which was reduced by 35% compared to a fully permeable membrane. The reduction in the nucleus yield for impermeable nuclear membranes results in a reduction to nuclear DNA, the primary biological target. However, the reduction in DNA damage may be offset by enhanced damage to other targets such as the nuclear membrane. The highly clustered absorption of reactive species in the membrane has the potential to cause holes in the membrane allowing oxidative species within the cytoplasm to enter the nucleus and contribute to the biological damage. The distribution of absorbed reactive species in the nuclear membranes demonstrates the highly heterogeneous reactive species distribution from the GNPs. While the macroscopic effects are minimal hotspots of very high reactive species concentrations on the microscale occurs near GNP clusters which can cause major local biological damage.

It was determined that inter-cellular effects were minimal, the maximum yield in the adjacent cell was less than 2.5% that within the cell with GNPs and was often much less. This indicates that for proton therapy, GNPs result in a targeted enhancement essentially effecting only the cells to which GNPs are delivered.

This work provides quantitative data for the first time on how cluster size and distribution affects absolute radiolysis yield in cell components, the effects of membrane permeability and the distribution of reactive species stopped in the membrane and intracellular radiosensitisation effects. These results can aid future experiments and clinical trials of GNP enhanced proton therapy.

GNP enhanced proton therapy is a promising treatment offering improved biological effect within tumour cells with internalised GNPs combined with the excellent sparing of healthy tissue of proton therapy. Effective enhancement of damage to a cellular component can be achieved if the GNPs are designed to accumulate adjacent to the targeted component. Higher efficiency is also achieved with smaller GNP clusters. While the GNP enhancement of proton therapy may be less than that of conventional photon therapy, the limited range of the enhancement is beneficial for specifically targeting cancer and sparing adjacent healthy cells, allowing for potentially superior improvements in the therapeutic effect.

## Figures and Tables

**Table 1:** The dose, radiolysis yield at 1 µs, dose enhancement factor (DEF) and radiolysis enhancement factor (REF) at 1 µs within the nucleus (N) and cytoplasm (C) for GNP cellular distribution simulations. Key results are also summarised.

Simulation		Dose Per Proton (eV) (GNP)		Yield Per Proton (GNP)		DEF		REF		Main Results
		N	C	N	C	N	C	N	C	
Cluster size (nm)	100	5.30	63.6	0.537	5.66	1.07	1.17	1.08	1.17	200 nm clusters had yields 117% and 25% greater than for 500 nm clusters for nucleus and cytoplasm respectively
	200	4.63	64.1	0.459	5.73	1.07	1.15	1.10	1.14	
	500	2.17	50.7	0.212	4.59	1.16	1.19	1.17	1.19	
Cluster distribution	Near nucleus	4.63	64.1	0.459	5.73	1.07	1.15	1.10	1.14	Clusters near nucleus increased nucleus yield by 91% while reducing cytoplasm yield by 7% compared to a disperse distribution
	Whole Cytoplasm	2.47	68.4	0.240	6.15	1.09	1.18	1.12	1.17	
Number of Clusters	500	2.49	32.3	0.246	2.88	1.15	1.17	1.16	1.17	Dose and yield per GNP changed by less than 7% indicating a good scaling with GNP concentration
	1000	4.63	64.1	0.459	5.73	1.07	1.15	1.10	1.14	
	2000	9.21	126.5	0.913	11.3	1.13	1.16	1.14	1.16	

**Table 2:** Nanoparticle distribution, membrane permeability and proton parameters used in simulations.

Simulation	Cluster Diameter (nm)	Total Number of GNPs (10^4^)	Distribution Range from Nucleus (nm)	Nuclear Membrane Permeability (%)	Cell Membrane Permeability (%)	Proton Energy (MeV)
Effect of cluster distribution	200	10	1000, 2250	100	100	5
Effect of Cluster size	100, 200, 500	10	1000	100	100	5
Effect of GNP concentration	200	5, 10, 20	1000	100	100	5
Nuclear membrane absorption	200	10	1000	0	100	5
Effect in adjacent cell	200	10	1000	100	100	5
Cell membrane absorption	200	10	1000	100	0	5

## Supplementary Materials

Supplementary materials can be found at https://www.mdpi.com/1422-0067/21/12/4431/s1.

## Abbreviations

GNP	Gold Nanoparticle
EPR	Enhanced Permeation and Retention
LEM	Local Effect Model
WNP	Water Nanoparticle
LET	Linear Energy Transfer
